# Inflammation as a Driver of Prostate Cancer Metastasis and Therapeutic Resistance

**DOI:** 10.3390/cancers12102984

**Published:** 2020-10-15

**Authors:** Maddison Archer, Navneet Dogra, Natasha Kyprianou

**Affiliations:** 1 Department of Urology, Icahn School of Medicine at Mount Sinai, New York, NY 10029, USA; Maddison.Archer@mountsinai.org; 2 Department of Pathology and Laboratory Medicine, Icahn School of Medicine at Mount Sinai, New York, NY 10029, USA; navneet.dogra@mssm.edu; 3 Department of Genomic Sciences, Icahn School of Medicine at Mount Sinai, New York, NY 10029, USA; 4 Department of Oncological Sciences, Icahn School of Medicine at Mount Sinai, New York, NY 10029, USA

**Keywords:** metastasis, survival signaling, anoikis, EMT phenotype, treatment resistance, tumor microenvironment, vascularity, macrophages

## Abstract

**Simple Summary:**

Prostate cancer is the most common malignancy in men, with a high mortality rate when disease progresses to metastasis and therapeutic resistance. Evidence implicates inflammation as a driver of prostate cancer risk and has a significant impact on processes in the tumor microenvironment that facilitate progression to advanced therapeutically resistant disease. In this review, we discuss the sources of inflammation in the prostate, the functional contribution of the critical inflammatory effectors to prostate cancer initiation and metastatic progression, and the therapeutic challenges that they impose on treatment of advanced disease and overcoming therapeutic resistance. Full understanding of the role of inflammation in prostate cancer progression to advanced metastatic disease and tumor relapse will aid in the development of personalized predictive biomarkers and therapy to reduce the burden and mortality in prostate cancer patients.

**Abstract:**

Prostate cancer is the most common malignancy among men, and progression to metastasis and the emergence of therapeutically resistant disease confers a high mortality rate. Growing evidence implicates inflammation as a driver of prostate cancer development and progression, resulting in increased cancer risk for prostate cancer. Population-based studies revealed that the use of antinflammatory drugs led to a 23% risk reduction prostate cancer occurrence, a negative association that was stronger in men who specifically used COX-2 inhibitors. Furthermore, patients that were taking aspirin had a 21% reduction in prostate cancer risk, and further, long-term users of daily low dose aspirin had a 29% prostate cancer risk reduction as compared to the controls. Environmental exposure to bacterial and viral infections, exposure to mutagenic agents, and genetic variations predispose the prostate gland to inflammation, with a coordinated elevated expression of inflammatory cytokines (IL-6, TGF-β). It is the dynamics within the tumor microenvironment that empower these cytokines to promote survival and growth of the primary tumor and facilitate disease progression by navigating the immunoregulatory network, phenotypic epithelial-mesenchymal transition (EMT), angiogenesis, anoikis resistance, and metastasis. In this review, we discuss the sources of inflammation in the prostate, the functional contribution of the critical inflammatory effectors to prostate cancer initiation and metastatic progression, and the therapeutic challenges that they impose on treatment of advanced disease and overcoming therapeutic resistance. Growing mechanistic evidence supports the significance of inflammation in localized prostate cancer, and the systemic impact of the process within the tumor microenvironment on disease progression to advanced therapeutically-resistant prostate cancer. Rigorous exploitation of the role of inflammation in prostate cancer progression to metastasis and therapeutic resistance will empower the development of precise biomarker signatures and effective targeted therapeutics to reduce the clinical burden and lethal disease in the future.

## 1. Introduction

### 1.1. The Clinical Problem of Prostate Cancer

Prostate cancer is the most common malignancy in men, with one in six American men developing prostate cancer within their lifetime, it is estimated that 191,930 American men will be diagnosed with prostate cancer in 2020 [1]. Prostate cancer is highly treatable when diagnosed early. However, one in 33 prostate cancer cases will result in death and makes prostate cancer the second leading cause of cancer-related deaths in American men. Factors that increase risk of developing prostate cancer include family history of disease, age, body mass index (BMI), and ethnicity [1].

**Figure 1:**
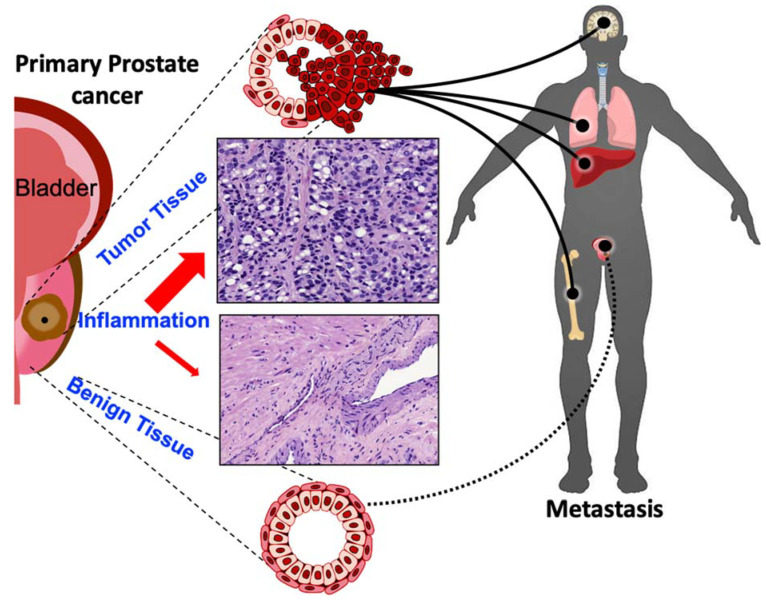
The Case of Inflammation within the Primary Prostate Tumors. Schema of normal prostate and corresponding/adjacent prostate tumor from biopsies in the patient. H&E staining indicating areas of inflammatory cell infiltration from the primary prostate cancer. Inflammation as a histopathological feature on the local prostate adenocarcinoma that remains organ-confined is not explicitly defined and its contribution in the context of navigating the aggressive behavior of prostate cancer to bone metastasis is even more poorly understood. We propose that inflammation in the primary tumor microenvironment can navigate the metastatic journey beyond the prostate including the liver, brain, lung, and bone. Differentiating prostate inflammation from cancer development early is critical to reducing overtreatment and overcoming therapeutic resistance.

The link between chronic inflammation and malignancies has been well established, and chronic inflammation has been identified as a major cause of approximately 20% of human cancer cases [2]. Inflammation in the prostate gland can be a result of several different contributing factors, such as viral or bacterial infection [sexually transmitted illness (STIs)], dietary habits, hormonal influences, urine reflux, or physical injury. However, while acute inflammatory reactions are critical for the clearance of pathogenic infection, sustained and uncontrolled inflammatory processes can lead to cellular and tissue damage. Chronic inflammation has been implicated in increasing the risk of developing malignancies, such as prostate cancer and tumor initiation. Further, chronic inflammation in the prostate microenvironment can alter the tumor microenvironment to favor cancer progression through proliferation, cell survival, evasion of immune surveillance, tissue remodeling, production of angiogenic factors, metastatic spread, and resistance to therapeutic agents (Figure 1).

In this review, we will discuss the current understanding of the role of inflammation in the prostate tumor microenvironment and progression to therapeutically resistant metastatic prostate cancer. Understanding the biological mechanisms by which inflammation contributes to prostate cancer progression may reveal targets for therapeutics to reduce the burden of prostate cancer in the future. Mechanistic insights into the influence/drivers of inflammation on the tumor microenvironment will enhance our knowledge regarding the contribution of this complex process of prostate cancer progression and therapeutic resistance.

### 1.2. Mechanistic Significance of Inflammation in Prostate Cancer

Inflammation is a critical immune process that occurs in response to tissue damage caused by injury or infection. Inflammatory processes aim to clear pathogenic material and debris from damaged tissue areas and initiate wound healing. While this process is an essential defense mechanism to fight invading pathogens, persistent chronic inflammation can cause a further tissue damage. This can result in the release of reactive oxygen and nitrogen species from cells, as well as increased genome instability leading to an increased risk of cancer [3]. When the cells are damaged, or there is an infection, the cells release agents to activate inflammatory signaling pathways, release inflammatory mediators and cytokines, recruit inflammatory immune cells, and increase vascular permeability [4]. One of the major inflammatory signaling pathways is elicited by NF-κB, a transcription factor that is predominantly activated during inflammation by cytokines, such as tumor necrosis-factor-α (TNF-α). Once activated, this transcription factor regulated expression of cytokines and factors involved in cancer development and progression such as IL-6 for tumor cell survival, angiogenic factors VEGF and IL-8, along with further production of inflammatory mediators for immune cell recruitment [5]. NF-κB activation occurs in many types of cancer, including prostate cancer and correlated with prostate cancer survival, progression, chemoresistance and metastasis [6].

Inflammation in early prostate carcinogenesis can be histologically indicated by proliferative inflammatory atrophy (PIA) (Figure 1), which are lesions containing activated inflammatory immune cell infiltrates within the peripheral zone of the prostate, where most cancers occur [7]. These lesions have a greater proliferation, potentially in response to cellular damage that is caused by inflammation, upregulation of apoptosis regulator Bcl-2, and the expression of proto-oncogene MYC [8]. This has been identified as precursor to prostatic intraepithelial neoplasia (PIN) and morphological studies have demonstrated that there is a transition from PIA to high grade PIN, benign prostate hyperplasia, and carcinoma [2]. The effects of anti-inflammatory use on prostate cancer risk, particularly non-steroidal anti-inflammatory drugs (NSAIDs), have demonstrated the significance of inflammation in prostate cancer. In a population-based case-control trial, prostate cancer occurrence was negatively associated with NSAID usage and a 23% risk reduction; this association was stronger in men who specifically used COX-2 inhibitors and consumed at least one pill per day [9]. Another analysis of aspirin use reported that men who currently use aspirin had a 21% reduction in prostate cancer risk and, further, long-term users of daily low dose aspirin had a 29% prostate cancer risk reduction when compared to those who did not take aspirin [10]. The ability of inflammation to modulate prostate cancer risk, suggests that it may play a significant role in the development of prostate cancer. A compelling body of evidence has identified diverse routes of inflammation within the prostate gland and causing prostatic diseases, infectious and non-infectious (as illustrated on Figure 1). These sources have the capacity to mediate sustained chronic inflammation and they can contribute to prostate cancer risk, development, and tumor progression to metastatic disease [11].

### 1.3. Origin of Inflammation 

#### 1.3.1. Infectious Causes of Inflammation

Bacterial Infections: bacterial infections can elicit an inflammatory response in the prostate leading to prostatitis; these infections include sexually transmitted infections (STIs). Previous studies have suggested that an STI diagnosis is more likely to exhibit an elevation in serum prostate specific antigen (PSA) and men with a history of diagnosis with an STI are more likely to develop prostate cancer [12,13]. However, it is important to note that not all STI’s progress to prostatitis, which is possibly due to treatment with antibiotics. Meta-analyses of epidemiological studies have reported that a history of infection with Neisseria gonorrhoeae is associated with an increased risk of prostate cancer, and this association was stronger in men of African American descent [12,14]. There is some evidence that syphilis infection is associated with an increased risk of prostate cancer in retrospective analysis; however, conflicting results have been reported in a prospective investigation conducted by Sutcliffe et al., which found no association between gonorrhea, chlamydia or syphilis diagnosis and prostate cancer [15,16,17]. In vitro studies have observed that Trichomonas vaginalis can induce an inflammatory response from prostate epithelial cells [18]. In case-control trials, it has been reported that men with antibodies against T. vaginalis were more likely to develop prostate cancer [19].

Viruses-Link to COVID19: viral infections are another potential source of local inflammation within the prostate; however, this area is not well understood, and it is not as extensively studied as bacterial infections. There is conflicting evidence regarding the relationship between prostate cancer and some viruses infecting the prostate. For example, in a case-control study there was no relationship found between human papillomavirus (HPV) infection and symptoms of prostatitis [20]. However, meta-analyses have reported there is an increased rate of prostate cancer in men with a history of HPV infection [12]. Previous studies have detected the herpes simplex virus (HSV) in prostatic fluid from patients exhibiting symptoms of chronic non-bacterial prostatitis, suggesting that infection with HSV can be associated with prolonged inflammatory symptoms in the prostate [21]. However, case-control studies suggest that there is no association between herpes simplex virus type 2 (HSV2) and human herpes virus type 8 (HHV8) serology with prostate cancer, while some meta analyses have reported an association of HSV2 with prostate cancer risk [22,23]. These studies demonstrate that the relationship between viral infections, local inflammation, and prostate cancer is unclear, and further investigation is required in order to understand this relationship.

COVID-19 Link: while there is little evidence that viral infections lead to inflammation within the prostate glandular tissue, systemic inflammation that is driven by viral infection easily creates a “favorable” environment for tumor development in various organs, including the prostate. The severe acute respiratory syndrome coronavirus 2 (SARS-CoV-2) pandemic that erupted in December 2019, led to the novel coronavirus disease (COVID-19), which has resulted in a global pandemic with over 31,890,248 people infected worldwide (https://coronavirus.jhu.edu/map.html, 20 September 2020) and more than 205,000 deaths in the United States. The available antibody tests checking for an antibody response to SARS-CoV-2 infection are used in order to determine virulence of infection and case fatality rates in COVID-19 patients. The mechanism of viral SARs infection induces an increase in circulating pro-inflammatory cytokines, including IL-6, IL-8, TGF-β, CCL2, and TNFα as compared to uninfected controls, and this elevation is sustained through viral clearance and recovery in patients [24,25]. Remarkably, there is a wealth of evidence that these cytokines contribute to the development and progression of prostate cancer [26]. The activation of IL-6 signaling can drive growth, proliferation and migration of prostate cancer cells [27]. Elevated serum levels of IL-6, IL-8, TNF-α, and CCL2 are associated with accelerated progression and poor prognosis in prostate cancer patients [28,29]. TGF-β is a pleiotropic cytokine, which has several roles in prostate cancer development and progression including proliferation, EMT, angiogenesis, invasion and metastasis [30]. Further, recent meta-analyses in COVID-19 patients reported that men had higher levels of immune cytokines that were associated with poor outcomes, such as TNFSF13B, CCL14, CCL23, IL-17, IL-16, and IL-18 [31]. Therefore, the systemic upregulation of these inflammatory cytokines could contribute to prostate cancer risk and exacerbate progression of prostate cancer to more aggressive disease.

The mechanism by which SARS-CoV-2 is internalized into target cells is facilitated by the activation of the type II transmembrane serine protease TMPRSS2 [32]. Interestingly, the expression of the TMPRSS2 gene is regulated by androgen signaling [33]. The TMPRSS2 gene has been found to form fusions with transcription factors, the TMPRSS2-ERG gene fusion is present in ~50% of prostate cancer cases [34,35]. The presence of the TMPRSS2-ERG gene fusion may lead to more aggressive disease [35,36]. These gene fusions have been observed to be a result of oxidative stress driven by inflammation that is mediated by TNF-α [37]. Thus, one may argue that the systemic inflammatory response induced by COVID19 infection could promote gene rearrangements to form TMPRSS2-ERG fusions and increase risk of prostate cancer. Growing evidence amid the pandemic implicates a role for inflammation as an underlying process contributing to both diseases, and a relationship to androgen signaling through TMPRSS2 [26]. Therefore, there is potential for overlapping use of therapeutics for both prostate cancer and COVID19. For example, anti-androgen therapy commonly used in prostate cancer has the potential for use to block the entry of COVID19 to target cells through attenuation of TMPRSS2 expression [38]. Other therapeutics that target inflammation that exacerbate COVID19 infection are currently being investigated, such as IL-6 inhibitors, which have also been studied for potential treatment of prostate cancer [39].

#### 1.3.2. Non-Infectious Sources of Inflammation

Sex Hormones: androgens and androgen receptor signaling is vital in the normal functioning of the prostate gland and regulating proliferation of prostate epithelial cells. The interplay between androgen and inflammatory signaling may influence the prostate microenvironment and the onset of prostate tumorigenesis. As men age, there is a decrease in serum androgen levels, while there is also an increase in the levels of circulating inflammatory cytokines, including TNF-α, IL-1β, and IL-6, along with an increase in anti-inflammatory IL-10 [40]. Systemically, androgens have a dampening effect on inflammatory mediators, and regulate the expression of TNF-α, IL-1β, IL-6, and C-reactive protein (CRP) [41]. The androgen receptor (AR) expression can interfere with the tumor suppressive effects of TGF-β during early stage of tumor development [30]. The blocking of AR signaling can drive increased inflammation in prostate cancer cells by the induced expression of CCL2, promoting prostate cancer progression via activation of the STAT3 signaling pathway and recruitment of TAMs [42]. AR signaling can block the canonical NF-κB pathway; therefore, the disruption of AR can lead to increased inflammatory markers [5]. Recent studies have reported that IL-1 expression by prostate cancer cells is inversely correlated with AR activity and androgen deprivation can induce production of IL-1, leading to a pro-inflammatory tumor microenvironment [43]. Thus, the collective evidence is pointing to the suppression of androgen signaling, sequentially leading to chronic inflammation.

Diet: dietary habits have been implicated as a source of inflammation towards driving an increased risk for prostate cancer. The process of cooking meat or fish past the point of smoke production, also results in the production of heterocyclic amines that might be carcinogenic [44]. Significantly enough, epidemiological evidence suggests that dietary heterocyclic amines are associated with an increased risk of breast and colorectal cancers [45,46]. Further epidemiological studies indicate a link between intake of overcooked meals with risk of prostate cancer [47,48]. In a study in male rats, it was reported that exposure to the most prevalent heterocyclic amine compound 2-amino-1-methyl-6-phenylimidazo[4,5-b] pyridine (PhIP) resulted in both intestinal and prostate carcinomas [49]. This increase in cancer risk may be associated with inflammation, another study reported that rats that were treated with dietary PhIP exhibited local inflammation and atrophy in the prostate preceding the development of PIN and intraductal carcinomas, along with an increased infiltration of inflammatory cells, such as macrophages and mast cells [50,51]. Rats that were exposed to PhIP had an increase in invasive cancers and decreased survival when infected with bacterial prostatitis. Further, these rats had elevated serum levels of inflammatory cytokines IL-6 and IL-12 [52]. Therefore, the risk of prostate cancer induced by PhIP may also be confounded by other risk factors, including bacterial prostatitis [52].

Studies have reported a positive relationship between obesity and high fat diet (HFD) and advanced prostate cancer, biochemical recurrence, and prostate cancer mortality. Obesity leads to chronic systemic inflammation, with greater circulating inflammatory cytokines such as TNF-α, leptin, CRP, MIC1 and IL-6 [53,54]. In the prostatic tissues of overweight patients, there is greater mRNA expression of IL-1, IL-6, and TNF-α when compared to patients with BMI < 25 [55]. Animal models have demonstrated that diet and obesity induced inflammation promotes prostate carcinogenesis. Mice with LNCaP prostate cancer cell xenografts exhibited increased circulating inflammatory factors MIC1 and CCL2, and greater tumor growth when administered HFD [53]. In a study utilizing the transgenic adenocarcinoma of mouse prostate (TRAMP) model, HFD promoted invasion and of prostate tumors and accelerated progression to metastasis when compared to control diet. The serum from these mice increased proliferation, invasion and migration of prostate cancer cell lines in vitro [56]. In studies using prostate specific *Pten* knockout mice, HFD-fed mice had greater serum IL-6 and tumor proliferation, and increased ratio of M2/M1 infiltrating macrophages. The attenuation of the diet-induced inflammation by COX-2 inhibitor celecoxib nullified the increase in tumor growth in HFD-fed mice [57]. Taken together, this evidence strongly suggests that HFD and obesity have a profound effect on the prostate cancer development and progression of prostate cancer to aggressive, advanced disease. Thus, we could confidently submit that a carefully designed balanced dietary regimen impacting inflammation within the tumor microenvironment may significantly improve clinical outcomes (treatment response, survival) of prostate cancer patients.

Trauma to Prostate Epithelium: Chemical irritation to the prostate epithelium can result in the release of pro-inflammatory cytokines and development of chronic inflammation, this can occur as a result of urine reflux [58]. Studies in rats have demonstrated that urine reflux leads to the infiltration of inflammatory cells and increased production of inflammatory cytokines IL-1α, IL-1β, IL-6, and TNFα in the prostate [59]. It is suggested that this is mediated through uric acid, which acts as a danger signal to activate the NALP3 inflammasome in innate immune cells, such as macrophages, leading to the infiltration of immune cells and production of inflammatory cytokines [60]. This can lead to further cellular damage and the formation of corpora amylacea [61]. Corpora amylacea is associated chronic with inflammation, pro-inflammatory factors, such as increased expression of COX-2, and it is common in men with prostate cancer [2,62].

Genetic Predisposition: a growing body of evidence has identified several genes and their variants involved in inflammation and immune function, which are linked to increased prostate cancer risk. The most studied gene linked to prostate cancer susceptibility is ribonuclease L (RANSEL), which encodes an enzyme that is induced by interferon that degrades viral RNA and modify innate immune responses [63]. Several mutated alleles that inactivate RNASEL, including E265X, Met1Ile, and GLU256X, are associated with prostate cancer among families, as well as 1623A > C and M1I in African family lines [64,65]. One RNASEL gene variant has been implicated in as much as 13% of prostate cancer cases, and men who are homozygous for the Arg46sGln allele mutation can exhibit double the risk of developing prostate cancer when compared to men who carry no RNASEL mutated alleles [66,67]. Furthermore, several mutations in the MSR1 gene can be predictive of increased inheritable risk of developing prostate cancer linked to inflammation [68]. This gene encodes a subunit of the macrophage scavenger receptor and facilitates inflammatory phenotypes in macrophages via JNK signaling [69]. However, there is controversy surrounding the association between MSR1 variants and prostate cancer risk. While evidence found no association between alterations in the MSR1 gene and prostate cancer [70,71,72], other studies reported that the ARG293x mutation in the MSR1 gene was detected in 2.5% of prostate cancer cases, and 0.4% in those without prostate cancer. This suggests that MSR1 mutations may only confer a moderate increase in prostate cancer risk, particularly in black men [73,74].

The macrophage inhibitory cytokine 1 (MIC1) is a member of the TGF-β superfamily that modulates macrophage mediated inflammation and is expressed by prostate cancers, and circulating levels can be linked to disease outcomes [75]. Studies using mouse models of prostate cancer have reported that the deletion of the MIC1 gene leads to increased primary tumor growth in early development [76]. A case control study reported that non-synonymous mutation in the MIC1 gene (H6D) is associated with prostate cancer [77].

Glutathione S-transferase protects against cell damage from oxidative stress, which can be a result of inflammation [78]. Methylation or other dysregulation of the glutathione S-transferase (GSTP1) gene and the subsequent loss of GSTP1 expression is found in 70–80% of all prostate cancer cases [79]. Epigenetic changes to GSTP1 is considered to be a marker for early prostate carcinogenesis [80]. Modifications to genes that are involved in inflammatory pathway hold the potential to influence both inherited and non-inherited prostate cancer risk. However, this is a large area of research and more work is required to elucidate the role of inflammation genetic variation and prostate cancer risk.

### 1.4. Inflammation Navigates the Primary Tumor Microenvironment

An inflammatory reaction is constantly elicited inside a malignant tissue, which results in an extraordinary dynamic of immune cell infiltration, angiogenesis, and fibroblast proliferation [81]. The tumor microenvironment is a dynamic network of cells and structures, including the tumor cells, the surrounding stroma that consists of cancer associated fibroblasts (CAFs), immune cells, adipocytes, mesenchymal stem cells (MSCs), extracellular matrix (ECM), as well as cytokines, chemokines, and growth factors secreted by these cells. The crosstalk between the cells, soluble and insoluble factors with cancer cells results in changes to the tumor microenvironment that regulate and contribute to a more aggressive phenotype.

Prostate cancer cells produce several inflammatory factors, which modify the tumor microenvironment in order to contribute to tumor cell growth, survival, invasion and progression. Prostate cancer cells and surrounding prostate stroma cells express high levels of inflammatory cytokines including interleukins IL-1, IL-6, and IL-8 in order to promote prostate tumor proliferation and survival [82,83,84]. IL-8 is primarily produced by prostate stromal cells and infiltrating macrophages to promote proliferation and inhibit apoptosis via the activation of the STAT3/AKT/NF-κB pathways in prostate cancer cells [85]. IL-6 has been well studied for its multiple roles in cancer, it is produced by prostate tumor cells, tumor-associated macrophages (TAMs), CD4+ T cells, and fibroblasts. IL-6 promotes tumor cell proliferation by acting through the STAT3 pathway to upregulate Myc expression and induces the expression of anti-apoptotic genes encoding bcl2, bcl-XL and survivin to promote tumor survival [86,87]. Further, tumor necrosis factor (TNF) is a key cytokine in inflammation that induces the nuclear factor kappa B (NF-κB) pathway in order to promote cancer cell survival via upregulation of STAT3.

Immunity: it is becoming increasingly evident that inflammation induces cancer development and resistance to therapy. Recent single cell analyses studies have revealed key insights into the Immune cell regulation and its contribution towards inflammatory microenvironment. In a recent study, Trujillo et al. discussed tumor segregation by T-cell inflamed phenotype and compared the genomic determinants of T-cell inflamed versus T-cell non-inflamed tumors [88]. This study revealed predictive responsiveness to therapeutic approaches, which can be precisely based on the inflammation or non-inflammation of the tissue. Subsequent studies identified specific circulating inflammatory dendritic cells (DC) among all of the other mononuclear phagocytes revealing Pro-inflammatory CD14+ DC cells that correlate with disease activity in lupus patients [89]. Furthermore, recent flowcytometric analyses has revealed unique myeloid cell populations in acute inflammation in lung tissue [90]. In conclusion, several recent studies have revealed that immune regulation is an important aspect of inflammation that may contribute to metastasis and therapeutic resistance.

The production of inflammatory cytokines, such as chemokine ligand 2 (CCL2) and IL-8, lead to an infiltration of immune cells such as macrophages. TAMs are a prominent cell type in the tumor microenvironment and they are associated with a poor prognosis in many types of cancers, including prostate cancer [91]. These cells modulate immune surveillance, and suppress anti-tumor immune responses by expression of IL-10 and transforming growth factor beta (TGF-β). TGF-β is a key player in mediating the inflammatory tumor microenvironment. TGF-β is a multifunction cytokine that is produced by prostate cancer cells and other cell types in the tumor microenvironment and has multiple roles in tumor development and progression. TGF-β facilitates cancer cells evading immune surveillance by inhibiting effector T cell immunity, inducing regulatory T cells (Treg) and further driving the differentiation of macrophages towards an TAM phenotype to sustain tumor immune suppressive functions [92,93].

Angiogenesis: angiogenesis is a vital process during tumorigenesis for providing homeostasis to the tumor microenvironment. Inflammation plays a supportive role in facilitating this process, TNF in the microenvironment stimulates the release of pro-angiogenic factors IL-6, IL-8, TGF-β, and vascular endothelial growth factor (VEGF) by cancer cells via the induction of NF-κB [94]. The inflammatory recruitment of TAMs also supports angiogenesis as they have high expression of factors to promote vascularization, including VEGF, TGF-β, platelet derived growth factor (PDGF), and basic fibroblast growth factor (bFGF) [95]. The release of TGF-β, IL-6, and bFGF primes the surrounding stromal cells in order to support tumorigenesis by ensuring the ECM is dynamic and allows for the invasion and migration of the tumor. TAMs secrete a series of matrix metalloproteinases (MMP-2, MMP-9), which function to remodel the ECM through enzymatic breakdown of the basement membrane and rearrangement of the collagen network in the stroma. This stromal remodeling not only supports the formation of blood vessel during angiogenesis, but also allows for the migration of tumor cells, as they adopt a highly invasive phenotype and journey to metastasis.

**Figure 2:**
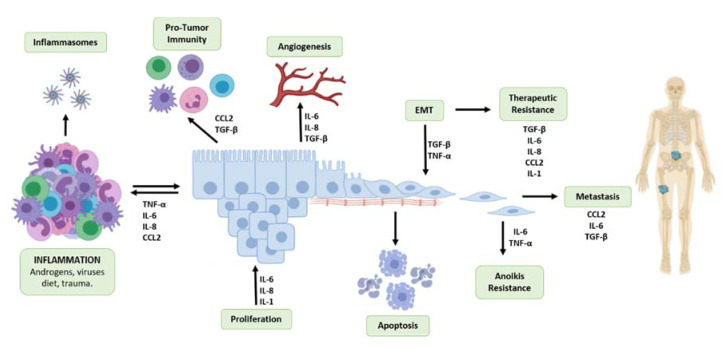
Overlapping functions of Inflammatory Cytokines Determine the Prostate Tumor Microenvironment Landscape and Metastatic Progression. Systemic and local inflammation in the prostate as a result of androgens, viral infections, trauma and diet is characterized by production of pro-inflammatory cytokines. In the microenvironment of primary prostate tumors, inflammation is sustained by production of pro-inflammatory cytokines by caner epithelial cells. These cytokines promote tumor cell proliferation, act as chemoattractants for tumor-associated macrophages (TAMs) and other immune cells to promote immune evasion, angiogenesis and ultimately tumor cell invasion and progression to metastasis to the bone. transforming growth factor beta (TGF-β) and tumor necrosis-factor-α (TNF-α) mediate the phenotypic reprogramming of tumor cells via Epithelial–Mesenchymal Transition (EMT) allowing the cells to lose their tight junctions and promote anoikis with consequential loss of attachment to basement membrane. This phenotypic reprogramming, along with influence of inflammatory cytokines confers resistance to therapeutics, via impacting cell survival as tumor cells are subjected to apoptosis; NF-κB signaling facilitates resistance to anoikis in tumor cells empowering migration, invasion and development of metastatic lesions primarily in the bone with the functional contributions by chemokine ligand 2 (CCL2), IL-6, and TGF-β within the bone microenvironment.

Epithelial–Mesenchymal Transition (EMT): inflammatory signals confer more invasive properties of a tumor by promoting the process of epithelial to mesenchymal transition (EMT) in cancer cells. Cancer cells adopt a mesenchymal cell phenotype by losing the cellular polarity and adhesion to the basement membrane to become more invasive and migratory (Figure 2). TGF-β has been identified as a key regulator of EMT in cancer, by the polarization of TAMs and activation of CAFs to induce the NFκ-B pathway and produce hypoxia-inducible factor (HIF-1) to drive expression of EMT proteins [96]. Inflammatory signals, such as TNF-α and TGF-β produced by immune cells in the tumor microenvironment, can induce the process of EMT in cancer cells by stimulating expression of proteins associated with EMT, including Snail, Zeb1, and Twist, leading to the degradation of adhesion molecules such as E-cadherin [97,98]. However, the role of TGF-β in EMT is functionally complex, and studies have reported that the disruption of TGF-β signaling can affect EMT by reducing E-cadherin expression and upregulated N-cadherin and Snail expression in mouse models of prostate cancer [30]. The upregulation of certain EMT protein effectors such as Snail can lead to sustained inflammation in the tumor microenvironment by the induction of IL-1, IL-6, IL-8, and cyclooxygenase-2 (COX-2) expression [96,97]. The expression of markers of EMT, such as vimentin and cytokeratin 8, are associated with poor prognosis is prostate cancer [99].

### 1.5. Impact of Inflammation on the Metastatic Journey

While the prognosis for most men with prostate cancer is good, those who develop advance metastatic castration resistant prostate cancer (mCRPC) have poor survival outcomes. Reliable prognostic markers would be useful as measures to predict prostate cancer risk, patient cancer progression, treatment response, and survival outcome. The use of inflammatory markers has been investigated as prognostic tools due to contribution of inflammation to prostate carcinogenesis. In a meta-analysis of over 16,000 men the neutrophil-lymphocyte ratio (NLR) was associated with poor overall survival, progression-free survival, and recurrence-free survival in men with prostate cancer and mCRPC irrespective of tumor stage and treatment [100]. Moreover an independent study on meta-analysis in men with mCRPC also revealed that elevated NLR was an independent predictor of poor prognosis [101]. Absolute neutrophil counts (ANC) have been reported to predict overall survival and prognosis in localized prostate cancer [102]. Other cell ratios analyzed towards identifying their prognostic predictive value is the platelet-lymphocyte ratio (PLR); it has been reported that elevated PLR is associated with poor disease free survival and overall survival in prostate cancer patients; significantly enough, this biomarker value was independent with ethnicity or tumor stage [103]. Conversely, a population based study reported that circulating blood counts of NLR and PLR were not associated with prognosis in prostatectomy patients [104].

Other circulating inflammatory factors that have been analyzed in various cancers for association with prostate cancer prognosis are CRP and lactate dehydrogenase (LDH) [105,106,107,108]. CRP is a standard circulating marker that is measured to detect both acute and inflammation in the body. LDH is an enzyme that is involved in cell damage, death, inflammation, and hemolysis [109]. Lactic acid levels are indicative of a hypoxic environment in cancer cells, a relationship of serum LDH with cancer progression has been considered [110]. Mounting evidence supports elevated serum LDH in various cancers as a potential predictive marker of treatment response [108,111]. Several studies have shown that these markers can predict treatment response and survival outcomes in patients with prostate cancer [100,103]. Large body of studies demonstrate that high CRP is negatively correlated with prognosis in metastatic prostate cancer patients, but it is not a prognostic predictor in localized disease [105]. Meta-analysis indicated that increased serum LDH was an independent prognostic factor of overall survival in both patients with CRPC and hormone sensitive prostate cancer [112]. A recent study that combined circulating blood cell ratios with CRP and LDH in order to create an inflammatory index as a prognostic tool in mCRPC, concluded that this combination inflammatory index is a viable prognostic predictor in patients with mCRPC.

Circulating inflammatory cytokines have been interrogated with precision in order to establish the relationship between inflammation and prostate cancer. Perhaps, the most well studied in cancer is IL-6, as a marker of chronic inflammation. Serum levels of IL-6 are elevated in patients with metastatic and CRPC and, IL-6 significantly correlated with tumor stage, and it is inversely correlated with tumor survival and therapeutic response [28,83,113]. TNF-α is a key mediator of inflammation; studies have demonstrated that serum TNFα is elevated in patients with metastatic prostate cancer, further serum TNF-α activity was positive in 76% of patients with relapsed disease and patients with elevated TNF-α has a higher mortality rate [113,114]. In a retrospective study, a series of inflammatory cytokines and chemokines were analyzed for their relationship with prostate cancer and progression to advanced disease. This study identified that elevated serum levels of inflammatory cytokines IL-8, TNF-α, and CCL2 were associated with accelerated progression to castration resistant disease and correlated with poor overall survival in prostate cancer patients [29]. The relationship between inflammatory markers and tumor progression place inflammation in the driver’s seat for prostate cancer tumorigenesis. Ongoing studies focus on defining the prognostic value of inflammatory markers in predicting disease outcomes.

Prostate cancer primarily metastasizes to the bone, and metastatic prostate cancer has a reduced five-year rate of survival of 30% from 99% in localized prostate cancer cases [1]. Inflammatory cytokines play a role in facilitating different stages in the metastatic process. Previously, we discussed the inflammatory contributors to processes in the tumor microenvironment that confer the invasiveness of cancer cells. These processes include EMT, which leads to the detachment of tumor cells from the ECM and surrounding cells, as well as angiogenesis. Here, we discuss the contribution of inflammatory signaling to further processes contributing to the metastatic journey of prostatic tumors, including resistance to anoikis cell death, migration, invasion, and colonization at the metastatic site.

As tumor cells undergo EMT, they adopt more invasive properties, lose their cell-cell adhesions, and detach from the ECM. Following detachment, cells are subject to anoikis, a form of cell death, which occurs in the absence of connections between the cells and ECM. However, tumor cells that develop resistance to anoikis can migrate freely in the circulation, leading to metastasis (Figure 1), contributing to therapeutic resistance, the development of mCRPC, and ultimately cancer patient mortality [115,116]. Studies in prostate cancer have demonstrated that the inflammatory NF-κB signaling pathway is constitutively activated, primarily by TNF, and it aids in resistance to apoptosis by inducing expression of anti-apoptotic proteins such as Bcl-xL [117]. NF-κB promotes tumor cell survival and anoikis resistance by activating the PI3K/Akt signaling cascade, and the downregulation of pro-apoptotic protein expression. Further, NF-κB signaling can promote the metastasis of cancer cells by regulating expression of Snail-1, IL-8, MMP-2 and -9, and VEGF to facilitate migration and invasion [118,119]. Other pathways, such as STAT3 signaling, can confer anoikis resistance when activated by inflammatory cytokines, such as IL-6, leading to the increased expression of pro-survival genes, including surviving, Bcl2, and cyclin D [86]. Studies on pancreatic cancer cells have demonstrated that exposure to IL-6 leads to enhanced STAT3 signaling and anoikis resistance [120].

Upon acquiring resistance to anoikis, tumor cells are able to migrate and invade distant sites, as schematically illustrated on Figure 2. Inflammatory cytokines play a key role in facilitating the migration of tumor cells. The inflammatory chemokine CCL2 promotes the infiltration of TAMs, which aids in immune evasion and angiogenesis [121]. This cytokine not only acts as a chemoattractant for monocytes, but also for prostate cancer epithelial cells [122]. Bone marrow endothelial cells express high levels of CCL2 in order to recruit prostate cancer cells and TAMs to the bone, which is the primary site for prostate cancer metastases. Further, CCL2 induced rearrangement of actin filaments to support invasion [122]. Serum IL-6 also acts as an attractant for tumor cells, facilitating migration [123]. Further, in vitro studies have demonstrated that IL-6 treatment promotes motility, migration, and decreases the adhesion of prostate cancer cells [124]. TNF-α promotes the migration of prostate cancer cells to the lymph nodes through the induction of CCR7/CCL21 interactions that promote chemotaxis through lymphatic tissues [125]. The action of TGF-β in prostate cancer metastasis is multilayered and complex, as this cytokine serves functionally opposing roles: as a master regulator of EMT within the tumor microenvironment, conferring tumor cell invasion and migration, while the loss of TGF-β signaling in prostate cancer in transgenic mouse models accelerated progression and metastasis to advanced disease [30,126]. On the other hand, during colonization release of TGF-β from the bone microenvironment promotes tumor growth in the bone and navigates the osteoblasts interaction with the colonized tumor cells in the bone microenvironment and development of metastatic lesions [127]. Significantly enough, cancer epithelial cells in the bone produce several inflammatory factors, such as IL-6, IL-8, TNF-α, and CCL2, which continue to promote immune evasion and interfere with normal bone forming and resorption processes [128]. (Figure 2)

Gene set enrichment analysis (GSEA), revealed that gene signatures that were related to interferon- and inflammatory cytokines signaling as well as apoptosis were the most significantly associated with the gene expression profile of AR-negative cancer epithelial cells, with an inverse association with DNA repair-associated gene signatures [129]. Moreover, The STING pro-inflammatory signaling cascade activated by the loss of AR function is a critical determinant of tumor infiltration by immune cells [130]. Increased cancer cells recognition and elimination by the immune system can be highly beneficial in a subset of melanoma patients [131].

### 1.6. Inflammation as a Contributor to Therapeutic Resistance

As prostate cancer progresses, there is emergence of androgen-independent state and tumors leading to metastatic castration-resistant prostate cancer, mCRPC. Patients who progress to this advanced disease have poor survival outcomes and they are highly resistant to therapeutic modalities, resulting in treatment failure, tumor recurrence, and patient lethality [132].

Androgen-deprivation therapy (ADT): growing evidence supports the role of inflammatory pathways in promoting progression to androgen-independent mCRPC disease. Recent studies have revealed that IL-1 represses activity of AR in prostate cancer cells and promotes the progression of prostate cancer to androgen-independent disease [43]. IL-23 produced by myeloid-derived suppressor cells (MDSCs) stimulate AR in prostate cancer cells in order to promote survival and proliferation during androgen deprivation therapy (ADT) and, therefore, promote progression to CRPC. Further, resistance to ADT can be overcome by inhibition of IL-23, there is potential for the use of anti-IL-23 antibodies to treat men with CRPC [133]. Several studies have implicated TGF-β signaling in progression to androgen independence in prostate cancer, dysregulated TGF-β signaling via dominant negative TGF-β type II receptor (DNTGF-βRII) in animal models of prostate cancer exhibit greater proliferation and reduced apoptosis following ADT. Further, AR and EMT regulator β-catenin were localized to the nucleus, suggesting that the crosstalk between TGF-β signaling, AR, and EMT mediate progression of prostate cancer to CRPC [134]. TGF-β may also regulate resistance to taxane chemotherapy; in vitro studies have demonstrated that prostate cancer cells that were treated with docetaxel exhibit greater survival when in the presence of TGF-β1 [135]. However, the complexity that surrounds the multifunctional roles of TGF-β in cancer development and progression, make it a challenging target for novel therapeutics. Recent studies reported that high expression of IL-8 in the prostate tumor microenvironment is associated with the loss of AR and aggressive disease [136]. Further, IL-8 expression is associated with resistance to conventional chemotherapy and targeting IL-8 can sensitize cancer to treatment [137]. IL-6 signaling has also been implicated as a potential inflammatory driver of therapeutic resistance in prostate cancer, with increased circulating IL-6 in patients with resistant disease [83]. LNCaP prostate cancer cells have reduced sensitivity to androgen depletion, when there is constitutive expression of IL-6 [138]. In line with this, prostate tumors that are resistant to ADT, or antiandrogen enzalutamide developed sensitivity to treatment when the IL-6/STAT3 signaling pathway was inhibited, which suggested that combination therapy of ADT and IL-6 antagonist may be promising in order to overcome this resistance [139]. Progression to androgen independence can be mediated by IL-6 and TNF-α activation of the NF-κB pathway; constitutive activation of NF-κB is associated with AR loss and castration resistant disease, the use of agents targeting this pathway has been suggested for therapeutics [140,141]. Further, the NF-κB pathway is activated by numerous chemotherapeutics, including docetaxel [142].

Taxane chemotherapy: taxane chemotherapy using microtubule-targeting drugs, such as docetaxel (1st line chemotherapy) and cabazitaxel (2nd line taxane chemotherapy), are standard modalities for the treatment for metastatic CRPC. Studies have investigated the ability of cytokines and macrophages to contribute to therapeutic resistance to taxane chemotherapy in prostate cancer patients impacting progression to lethal disease. In men with CRPC, elevated levels of circulating inflammatory cytokines IL-4, IL-6, and MIC1 were associated with resistance to docetaxel after one cycle of treatment [143]. Macrophage chemoattractant CCL2 has been implicated for involvement as an inflammatory driver of resistance to chemotherapeutics (Table 1). The inhibition of CCL2 has been demonstrated in order to improve sensitivity to docetaxel in prostate cancer cell in vitro and the overexpression of CCL2 can enhance cell proliferation in prostate cancer cells that were treated with docetaxel. This resistance is thought to be mediated through activation of PI3K/AKT survival pathway and inhibition of apoptotic proteins, such as Bcl2 [144]. Additional evidence suggests the potential for CCL2 inhibition as a target for overcoming chemotherapeutic resistance in prostate cancer bone metastases. This study used a mouse model of prostate cancer in the bone treated with combinations of docetaxel and CCL2 neutralizing antibodies, and reported that CCL2 neutralization inhibited metastatic prostate cancer lesions to the bone, an effect that is enhanced by combination with the first line taxane chemotherapy, docetaxel [145].

Radiotherapy: radiation therapy is commonly used for the treatment of localized prostate cancer. However, radiation resistance emerges and presents a therapeutic challenge among individual tumors [146]. Radioresistant prostate tumors depend on elevated DNA repair system and the intracellular levels of reactive oxygen species (ROS) to proliferate, self-renew, and scavenge anti-cancer regimens, whereas EMT enables radioresistant prostate cancer cells to metastasize after exposure to radiation. Clinicopathological analysis reveals that different regions within individual tumors have a gradient of radiosensitivity, depending on the microenvironment, inflammation, distribution of cancer stem cells, and gene profiles that confer radioresistance [147]. Prostate cancer radioresistance is conversely correlated with hypoxia within the tumor microenvironment that confers a radioresistant and metastatic phenotype of prostate tumors and, ultimately, disease recurrence after radiotherapy [148]. Among hypoxia-induced signals, extracellular vesicles called “exosomes” are the latest marker in mediating hypoxia-induced prostate cancer progression, potentially via tumor microenvironment remodeling [149]. Exosomes have been reported in hypoxia-induced angiogenesis, cancer stemness, activation of cancer-associated fibroblasts, and EMT [150]. The exosomes that are secreted under hypoxia enhance the invasiveness and stemness of prostate cancer cells by targeting adherent junction molecules and increased level of diverse signaling molecules (TGF-β2, TNFlα, IL-6, TSG101, Akt, ILK1, MMP, and β-catenin) [150]. Therefore, exosomes serve as vehicles that transfer bioactive molecules between cells and mediate cell-cell communication during hypoxia-derived radioresistance, enabling their utilization for monitoring disease and therapeutic response [151].

## 2. Conclusions and Future Directions

Detailed analyses of viral- and bacterial-infections, genetic variants, cytokines, and chemokines have paved an avenue for cornerstone studies to the next-gen prostate cancer diagnostics. In this context, future clinical trials should focus on the interventional studies in order to demonstrate the clinical utility of inflammatory markers in cancer.

Exposure to numerous infectious and non-infectious agents can promote inflammation; however, the link between this exposure and prostate cancer development and progression requires further analysis. Future studies should investigate the cellular and molecular changes that occur in individuals who have been exposed to inflammation inducing agents and how this may drive aggressive prostate cancer. The recent global COVID-19 pandemic provides a unique opportunity to examine how exposure to viral infections, and the resulting systemic inflammation and circulating inflammatory markers, can impact disease progression and outcomes in patients with cancer; this will be particularly in the context of prostate cancer due to the commonality of androgen signaling and TMPRSS2 involvement.

Progression to CRPC resistance and therapeutic resistance holds significant challenges to patient mortality; understanding the inflammatory signals that drive these processes can lead to effective therapeutic targeting by existing anti-inflammatory agents (as summarized on Table 1). Indeed, a decade ago, the first randomized, double-blind trials were conducted in order to examine the effect of COX2-inhibitors (Dexamethasone and Celecoxib) in prostate cancer patients [152,153]. One such study revealed a significant reduction in COX2 expression in tumor tissue, when compared with the adjacent benign tissue [152]. Of note, a higher expression of proliferation markers was another interesting outcome in these patients. Furthermore, a low dose combination (dexamethasone and celecoxib) therapy has been applied, which yielded significant long-term benefits in CRPC patient [153]. In contrast, combination Celecoxib and hormone therapy yielded no additional benefits [154]. Overall, the administration of low does Celecoxib (400 mg) twice daily for up to one year was not effective in patients starting hormone therapy for high-risk prostate cancer, and it was not recommend [152]. However, combination therapy may have a positive impact on Castration-Resistant Metastatic Prostate Cancer patients.

While several inflammatory cytokines have been implicated in disease progression and resistance, the mechanisms and pathways by which this occurs are not fully understood. Future studies should utilize both in vitro and in vivo models in order to target these inflammatory cytokines and examine the downstream effects on the expression of key factors that are involved in EMT, anoikis resistance, and metastasis. This may reveal more specific targets to combat the mechanism of resistance as the multifunctional roles of many inflammatory cytokines make them difficult to target safely in clinical situations. As the precise role of inflammation in the emergence of therapeutic resistance still needs to be exploited, identifying inflammatory signatures in different stages of prostate cancer progression and the impact of various therapeutic strategies on such signatures in patients with advanced prostate cancer may reveal critical mediators of therapeutic resistance and attractive new targets for overcoming this resistance (Table 1).

This review exploits the functional contribution of inflammatory cytokines to the development and progression of prostate cancer to metastasis, and the emergence of therapeutic resistance and treatment failure. Inflammatory cytokines have shared functions contributing to diverse processes that landscape the prostate tumor microenvironment and cancer progression to metastasis, as illustrated in Figure 2. However, the mechanistic involvement of inflammation as a causative event in prostate cancer initiation, beyond the phenotypic landscape, is still under interrogation. Understanding the functional relationship coordinating the inflammatory signatures in the tumor microenvironment may lead to better prediction of prostate cancer progression and response to therapeutic strategies in advanced disease, allowing for personalized medicine (targeted treatment strategies) towards reducing prostate cancer mortality.

## Figures and Tables

**Table 1:** Inflammation as a Developmental Platform for Predictive Signatures and Targeted Therapeutics in Advanced Prostate Cancer.

Inflammatory Factors	Therapeutics	Targeted Processes	Anti-Inflammatory Agents for Cancer
IL-1	IL-1 blockers (anakinra, canakinumab) [155]	Proliferation, survival, therapeutic resistance	NSAIDs- Aspirin, COX-2 inhibitors, Naproxen Sodium [9,10] Steroids- dexamethasone [156] Metformin [157] α1-adrenoceptor antagonists- quinazolines (doxazosin), sulfonamides (tamulosin) [158] Curcumin [159]
IL-6	Anti-IL-6/IL-6 receptor antibodies (Siltuximab, Tocilizumab) [123]	Proliferation, survival, anoikis resistance, metastasis, therapeutic resistance	
IL-8	Anti-IL-8 antibodies (HuMaxIL8) [160]	Proliferation, survival, angiogenesis, therapeutic resistance	
IL-23	Anti-IL-23 antibodies [133]	Therapeutic resistance	
NF-κB	Proteasome Inhibitors /Histone deacetylase inhibitors IKK inhibitors, Piperlongumine, Simvastatin [140]	Anoikis resistance, metastasis, therapeutic resistance	
CCL-2	CCL2 neutralizing antibody [145]	Pro-tumor immunity, metastasis, therapeutic resistance	
TGF-β	TGF-β receptor 1 inhibitor galunisertib [161]	Pro-tumor immunity, angiogenesis, EMT, metastasis, therapeutic resistance	
TNF-α	Anti-TNF antibodies (infliximab, etanercept) [162]	Survival, EMT, anoikis resistance,	

## Abbreviations

ADT	Androgen deprivation therapy
AKT	protein kinase B
ANC	Absolute neutrophil count
AR	Androgen receptor
Bcl-2	B cell lymphoma 2
bFGF	Basic fibroblast growth factor
BMI	Body mass index
CAF	Cancer associated fibroblast
CCL	Chemokine ligand
CCR	Chemokine receptor
CD	Cluster of differentiation
COVID-19	Coronavirus disease of 2019
COX-2	Cyclooxygenase 2
CRP	C-reactive protein
CRPC	Castration resistant prostate cancer
DNA	Deoxyribonucleic acid
DNTGF-βRII	Double-negative TGF-β receptor 2
ECM	Extracellular matrix
EMT	Epithelial-mesenchymal transition
ERG	ETS-related gene
GSEA	Gene set enrichment analysis
GSTP1	glutathione S-transferase
HFD	High fat diet
HHV8	Human herpes virus 8
HIF-1	Hypoxia inducible factor 1
HPV	Human papillomavirus
HSV	Herpes simplex virus
IL	Interleukin
JNK	c-Jun N-terminal kinase
LDH	Lactate dehydrogenase
LNCaP	Lymph node carcinoma of the prostate
M1I	Matrix protein 1
MDSC	Myeloid-derived suppressor cells
MET	Mesenchymal-epithelial transition
MIC1	Macrophage inhibitory cytokine 1
MMP	Matrix metalloproteinase
MSC	Mesenchymal stem cell
MSR1	Macrophage scavenger receptor 1
MYC	MYC Proto-Oncogene, BHLH Transcription Factor
NALP3	NACHT, LRR and PYD domains-containing protein 3
NFκB	Nuclear factor kappa beta
NLR	Neutrophil-lymphocyte ratio
NSAID	Non-steroidal anti-inflammatory drugs
PDGF	Platelet derived growth factor
PhIP	2-amino-1-methyl-6-phenylimidazo[4,5-b] pyridine
PI3K/Akt	Phosphoinositide 3-kinases/Protein kinase B
PIA	Proliferative inflammatory atrophy
PIN	Prostatic intraepithelial neoplasia
PLR	Platelet-lymphocyte ratio
PSA	Prostate specific antigen
RNASEL	Ribonuclease L
ROS	Reactive oxygen species
SARS-CoV2	Severe acute respiratory syndrome-coronavirus 2
STAT3	Signal transducer and activator of transcription 3
STI	Sexually transmitted illness
STING	Stimulator of interferon genes
TAM	Tumor associated macrophage
TGF-β	Transforming growth factor beta
TMPRSS2	Transmembrane protease serine 2
TNF	Tumor necrosis factor
TNFSF13B	Tumour necrosis factor superfamily member 13B
TRAMP	Transgenic adenocarcinoma of mouse prostate
VEGF	Vascular endothelial growth factor
